# Involvement of a putative intercellular signal-recognizing G protein-coupled receptor in the engulfment of *Salmonella* by the protozoan *Tetrahymena*

**Published:** 2013-07-06

**Authors:** P.N. Agbedanu, M.T. Brewer, T.A. Day, M.J. Kimber, K.L. Anderson, S.K. Rasmussen, M.A. Rasmussen, S.A. Carlson

**Affiliations:** 1 *Contributed equally to this work*; 2 *Department of Biomedical Sciences, College of Veterinary Medicine, Iowa State University, USA*; 3 *Veterinary Microbiology and Preventative Medicine, College of Veterinary Medicine, Iowa State University, USA*; 4 *Leopold Center for Sustainable Agriculture, Iowa State University, USA*

**Keywords:** GPCR, Protozoa, *Salmonella*, Virulence

## Abstract

In an effort to investigate the molecular basis of protozoa engulfment-mediated hypervirulence of *Salmonella* in cattle, we evaluated protozoan G protein-coupled receptors (GPCRs) as transducers of *Salmonella* engulfment by the model protozoan *Tetrahymena*. Our laboratory previously demonstrated that non-pathogenic protozoa (including *Tetrahymena*) engulf *Salmonella* and then exacerbate its virulence in cattle, but the mechanistic details of the phenomenon are not fully understood. GPCRs were investigated since these receptors facilitate phagocytosis of particulates by *Tetrahymena*, and a GPCR apparently modulates bacterial engulfment for the pathogenic protozoan *Entamoeba histolytica*. A database search identified three putative *Tetrahymena* GPCRs, based on sequence homologies and predicted transmembrane domains, that were the focus of this study. *Salmonella* engulfment by *Tetrahymena* was assessed in the presence of suramin, a non-specific GPCR inhibitor. *Salmonella* engulfment was also assessed in *Tetrahymena* in which expression of putative GPCRs was knocked-down using RNAi. A candidate GPCR was then expressed in a heterologous yeast expression system for further characterization. Our results revealed that *Tetrahymena* were less efficient at engulfing *Salmonella* in the presence of suramin. Engulfment was reduced concordantly with a reduction in the density of protozoa. RNAi-based studies revealed that knock-down of one the *Tetrahymena* GPCRs caused diminished engulfment of *Salmonella*. *Tetrahymena* lysates activated this receptor in the heterologous expression system. These data demonstrate that the *Tetrahymena* receptor is a putative GPCR that facilitates bacterial engulfment by *Tetrahymena*. Activation of the putative GPCR seemed to be related to protozoan cell density, suggesting that its cognate ligand is an intercellular signaling molecule.

## Introduction

Protozoa engulf *Salmonella* and, while the bacteria reside inside the protozoa, host cell invasion capabilities are hyperactivated and the bacteria are hypervirulent after exiting the protozoa. This protozoa-mediated hypervirulence occurs only for *Salmonella* strains bearing the multiresistance integron designated as SGI1, and its *in vivo* relevance has only been associated with protozoa that engulf *Salmonella* in the bovine rumen (Rasmussen *et al.*, 2005; Carlson *et al.*, 2007; Xiong *et al.*, 2010; Brewer *et al.*, 2011). Free-living protozoa, like *Acanthamoeba* and *Tetrahymena*, are models for studying this phenomenon (Carlson *et al.*, 2007; Xiong *et al.*, 2010; Brewer *et al.*, 2011).

Protozoal determinants of engulfment are unclear but Renaud *et al*. (1995) determined that G protein-coupled receptors (GPCRs) are apparently involved in *Tetrahymena* phagocytosis of particulates. Furthermore, Picazarri *et al*. (2005) demonstrated that bacterial engulfment is governed by a GPCR in the pathogenic protozoan *Entamoeba histolytica* (Picazarri *et al.*, 2005). Additionally, Lampert *et al*. (2011) presented evidence for GPCR-encoding genes in the *Tetrahymena* database (Lampert *et al.*, 2011).

The aim of this study was to assess the role of three putative GPCRs in the engulfment of *Salmonella* by *Tetrahymena*. These three putative GPCRs were identified in a GenBank database search. We used RNAi to preliminarily determine if any of these putative *Tetrahymena* GPCRs are involved in *Salmonella* engulfment. A heterologous yeast expression system was used to partially characterize one of these candidate *Tetrahymena* GPCRs. Using these approaches we were able to identify a putative *Tetrahymena* GPCR involved in the engulfment of *Salmonella*.

## Materials and Methods

### Tetrahymena culture

*Tetrahymena thermophila* was obtained from ATCC and axenically grown in the recommended ATCC medium (5.0 g/L proteose peptone, 5.0 g/L tryptone, and 0.2 g/L K2HPO4) at 25°C. Media was replaced every four days and cells were diluted 1:14 in fresh media.

### Tetrahymena engulfment of Salmonella in the presence of suramin

Approximately 10^5^ to 10^9^ CFUs/mL of bacteria were added to approximately 10^1^ to 10^5^ *Tetrahymena*/mL, maintaining a multiplicity of infection equal to 10^4^. The *Salmonella-Tetrahymena* mixture was then gently rolled for 16 hrs at 37°C in a sealed 5 mL glass tube, in the presence of various concentrations of suramin (0-200μM)- a non-specific inhibitor of GPCRs (Freissmuth *et al.*, 1999). At the end of the 16-hour incubation period, extracellular *Salmonella* were killed using 300 μg/mL florfenicol [Schering-Plough; (Rasmussen *et al.*, 2005)].

Protozoa were then lysed for 60 sec at 4,800 rpm using 2.5 mm glass beads and a mini-beadbeater (Biospec Products). The lysate was centrifuged at 10,000 x g for 2 min, then resuspended in 350 μL Lennox L broth (Difco) that was plated on *Salmonella*-selective XLD agar plates (Fisher Scientific) and then incubated at 37°C overnight. Characteristic black colonies were then counted the following day. Percent engulfment was calculated as such: 100x (number of *Salmonella* recovered from protozoa/number of *Salmonella* added to *Tetrahymena*).

### RNAi experiments

A preliminary GenBank database search identified three putative GPCR genes in *Tetrahymena* (XM_001009792.2, XM_001027519.2, and XM_001010055.2). siRNA was designed, using the Invitrogen webportal (*http://rnaidesigner.invitrogen.com/rnaiexpress/*), to silence expression of these three genes in *Tetrahymena*. The sequences GAGATTACTACTAATAGCCTCTCTT, GCTGATTCATTTAATAGCCTTGCTT, and TGGCTCAGTGTAAGTGACTTAATAT were deemed to be appropriate siRNA targets for the three putative GPCRs, respectively. A random sequence (CTGACGACAGTTGCATAAAGC) was used as a control siRNA. Semi-quantitative RT-PCR (Carlson *et al.*, 2007) was used to confirm the knock-down of the genes encoding the putative GPCRs (Table 1).

**Table 1:** Semi-quantitative RT-PCR of GPCR targets in *Tetrahymena* transformed with siRNA. *Tetrahymena* were transformed with siRNA and then DNA-free RNA was isolated and subjected to an RT-PCR assay that semi-quantitates transcript levels based on the number of PCR cycles required for amplicon visualization using agarose gel electrophoresis (Carlson *et al*., 2007).

GenBank accession numbers corresponding to the source of the siRNA used in *Tetrahymena*	Number of PCR cycles required for visualization of amplicons specific to the following GenBank sequences in *Tetrahymena* transformed with siRNA		
	XM_ 00100979.2	XM_ 001027519.2	XM_ 001010055.2
XM_00100979.2	50**	10	15
XM_001027519.2	15	>50**	10
XM_001010055.2	10	15	50**
random RNAi	15	10	15
mock RNAi	10	10	10

** on-target effects on transcription.

*Tetrahymena thermophila* were grown in 5 mL of media to reach confluency (3 × 10^4^ cells/mL), and were then harvested by centrifugation at 3,000 x g for 10 min. The pellet was washed twice in 15 mL of deionized water and resuspended in 200 μL of deionized water. These cells were then electroporated (0.2 cm electrode gap, 10 μF, and 5 milliseconds) with 5 nM siRNA.

At 24 hours after electroporation with siRNA, *Tetrahymena* cells were centrifuged at 4,000 x g for 5 min. *Tetrahymena* were then spectrophotometrically enumerated and resuspended in 1 mL of Luria-Bertani broth containing SGI1-bearing *Salmonella* at a multiplicity of infection equal to 10^4^. After 1 hr of co-incubation, non-engulfed bacteria were then killed with florfenicol (300 μg/mL) and protozoa were lysed with bead-beating. Protozoal lysates were recovered and plated on selective agar (XLD) for the enumeration of *Salmonella* engulfed by *Tetrahymena*, as described herein.

### Manipulation of the gene encoding the Tetrahymena GPCR

RNAi studies identified one of the putative GPCRs [XM_001009792.2] as a potential determinant of *Salmonella* engulfment.

In order to deorphanize this receptor in the yeast heterologous expression system, its gene was cloned into a yeast expression vector. Since *Tetrahymena* genes have read-through stop codons encoding glutamine residues (Adachi and Cavalcanti, 2009), the gene sequence encoding this GPCR was synthesized (GeneScript) whereby the 13 read-through stops codons (TAA or TAG) were exchanged for glutamine codons (CAA or CAG). Codons were also optimized for expression in yeast.

### Construction and cloning of the yeast expression vector containing the Tetrahymena GPCR gene

The synthetic *Tetrahymena* GPCR gene was PCR-amplified with forward (5’GCCATA*CCATGG*ACCAATCATTTGGAAATCAA3’) and reverse (5’GCCATA*GGATCC*TCAAGTTAGATTTATTTCACGTGAAT3’) primers, which included filler sequences (underlined) and the restriction sites *NcoI* and *BamHI* (italicized) incorporated into the 5’ and 3’ ends of the amplicon, respectively. Purified amplicons and the linearized yeast expression vector Cp4258, which bears a leucine prototrophic marker (Kimber *et al.*, 2009; Wang *et al.*, 2006), were co-digested with *NcoI* and *BamHI* restriction endonucleases (New England BioLabs).

The digested vector and amplicons were ligated with T_4_ DNA ligase (New England BioLabs) and the resulting plasmids were transformed into *Escherichia coli* K12 and individual clones were selected in ampicillin (resistance encoded by the Cp4258 vector) and grown aerobically in LB broth overnight at 37°C. Plasmid DNA was purified using the HiSpeed Plasmid Mini Kit (Qiagen) and inserts were verified using PCR and then sequenced to confirm gene orientation and fidelity.

### Transformation of yeast with the Cp4258/GPCR expression vector

*Saccharomyces cerevisiae* strain CY 19043 (J. Broach, Princeton University, USA) was used as the yeast recipient since these cells are leucine/histidine auxotrophs and exhibit a histidine prototrophic phenotype upon GPCR activation even if the receptor is exogenous (Wang *et al.*, 2006; Kimber *et al.*, 2009).

Non-transformed CY 19043 yeast were grown in YPD media supplemented with all essential amino acids. Cells at mid-log phase (OD_600_ equal to 0.3 to 0.5) were co-incubated with 1μg of Cp4258/GPCR construct, or Cp4258 bearing a non-*Tetrahymena* GPCR gene [an unpublished putative GPCR sequence from *Cryptosporidium parvum* (*C. parvum*)], in the presence of 200 μg salmon sperm DNA (Invitrogen) and 0.1M LiAc (Sigma-Aldrich).

Yeast cells were then incubated at 30°C and then heat shocked at 42°C for 15 minutes. Cells were plated on leucine-deficient media [1x YNB (Difco), 1x yeast synthetic dropout medium supplement without leucine (Sigma), 10mM ammonium sulfate (Sigma), and 50% glucose] to select for transformation of Cp4258/GPCR. Transformants were verified by isolating plasmids (Promega Wizard Prep kit) and PCR-based detection of the *Tetrahymena* GPCR gene insert and its proper orientation.

### Partial deorphanization of the putative Tetrahymena GPCR using the yeast heterologous expression system

A volume of 3 mL of leucine-deficient media was inoculated with yeast expressing the *Tetrahymena* GPCR, or the control, and grown at 30°C to an OD_600_ equal to one. Cells were washed three times with leucine/histidine deficient medium [1x YNB (Difco), 1x yeast synthetic drop out medium supplement lacking leucine and histidine (Sigma), 10mM ammonium sulfate, 50% glucose, 50mM 4-morpholinepropanesulfonic acid, pH 6.8], and then resuspended in 1 mL leucine/histidine-deficient media to a density of 15-20 cells/µL. Approximately 3,000 cells were added to each well of 96-well plates containing the same medium along with 50 µL of *Tetrahymena* culture media, *Tetrahymena* lysates (15-20 cells/µL lysed by bead-beating), or *Tetrahymena* lysates plus suramin (200μM). Cells were grown at 30°C for approximately 24 hours after which growth was measured spectrophotometrically at OD_600_. Yeast cells transformed with the *C. parvum* GPCR-encoding vector were used as a control for each treatment.

### Statistical analysis

Statistical comparisons were made using ANOVA with Scheffe’s F test for multiple comparisons. Comparisons were made across protozoal densities, suramin treatments, siRNA transformations, and yeast treatments. GraphPad Prism 5.0 was the software used, with *p*<0.05 indicating statistical significance.

### Hydropathy analysis of the putative Tetrahymena GPCR

Since GPCRs are comprised on seven transmembrane domains (Kobilka *et al.*, 1987), putative transmembrane domains were assessed using Kyte-Doolittle plots (Kyte and Doolittle, 1982) from a web-based platform (*http://web.expasy.org/cgi-bin/protscale/protscale.pl*). Transmembrane signatures were ascribed to peptide regions in which hydropathy scores rose from negative values to values greater than one. The codon-optimized deduced version of the protein, where glutamine codons replace read-through amber (TAG) or ochre (TAA) stop codons, was used for the hydropathy analysis.

## Results

### Suramin-mediated inhibition of the protozoa density-dependent engulfment of Salmonella by Tetrahymena

**Fig. 1:**
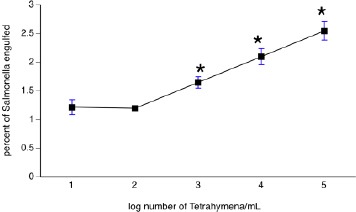
*Salmonella* engulfment at various densities of *Tetrahymena*. For each density of protozoa, *Salmonella* and *Tetrahymena* were co-cultured using a multiplicity of infection equal to 10,000 (*i.e*., bacteria:protozoa ratios of 10^9^:10^5^, 10^8^:10^4^, 10^7^:10^3^, 10^6^:10^2^, and 10^5^:10^1^). Bacterial engulfment (average number of bacteria engulfed per cell) was determined after lysing protozoa. Each data point represents the mean ± sem for three independent experiments each performed in triplicate. *p<0.05 versus the rest of the data.

**Fig. 2:**
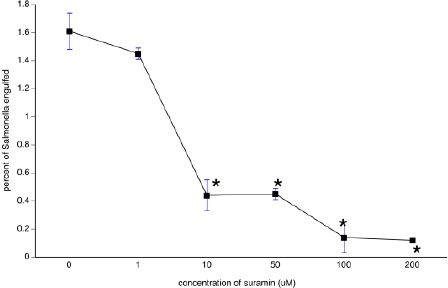
*Salmonella* engulfment by *Tetrahymena* exposed to suramin. For each concentration of suramin, *Salmonella* and *Tetrahymena* were co-cultured using a multiplicity of infection equal to 10,000 (*i.e*., bacteria:protozoa ratios of 10^9^:10^5^, 10^8^:10^4^, 10^7^:10^3^, 10^6^:10^2^, and 10^5^:10^1^). Bacterial engulfment (average number of bacteria engulfed per cell) was determined after lysing protozoa. Each data point presents the mean ± sem for the five different *Salmonella*:*Tetrahymena* ratios. *p<0.05 versus the results from suramin-free assays.

In order to assess the possible role of a GPCR in the engulfment of *Salmonella* by *Tetrahymena*, we incubated *Salmonella-Tetrahymena* co-cultures with the non-specific GPCR inhibitor suramin. As shown in Fig. 1, bacterial engulfment increased with the density of *Tetrahymena*. As shown in Fig. 2, this engulfment was significantly hampered by suramin in a concentration-dependent manner at all *Tetrahymena* densities.

### RNAi-based screening of a GPCR involved in bacterial engulfment by Tetrahymena

Our preliminary database query identified three *Tetrahymena* genes encoding putative GPCRs (XM_001009792.2, XM_001027519.2, and XM_001010055.2).

In order to assess the association of these GPCRs with bacterial engulfment, we knocked-down receptor expression with siRNA and then evaluated bacterial engulfment in *Tetrahymena*.

**Fig. 3:**
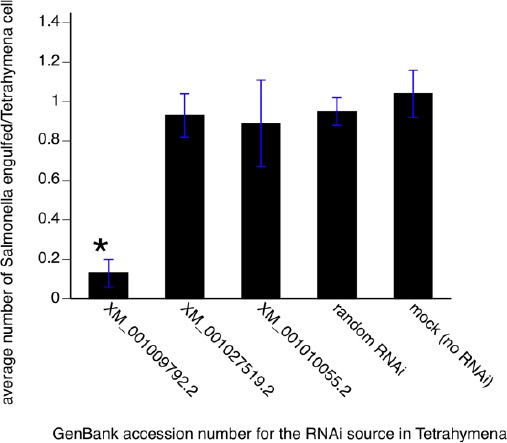
*Salmonella* engulfment by *Tetrahymena* electroporated with RNAi corresponding to putative GPCR genes. A preliminary database search identified three putative GPCR-encoding genes (GenBank accession numbers provided in the x-axis) in *Tetrahymena* and siRNA was designed based on these sequences. Bacterial engulfment (average number of bacteria engulfed per cell) was determined after electroporation with siRNAs. Random RNAi and mock transformants served as controls. Data presented are the mean ± sem for three independent experiments each performed in triplicate. *p<0.05 versus the rest of the data.

As shown in Fig. 3, bacterial engulfment was significantly hampered in *Tetrahymena* electroporated with one of the siRNAs corresponding to the gene with GenBank accession number XM001009792.2. Expression of this receptor was diminished as verified by semi-quantitative RT-PCR, and no off-target effects were noted in the transformants (Table 1).

### Partial deorphanization of the putative Tetrahymena GPCR using Tetrahymena lysates and suramin

Results presented in Fig. 3 identified a putative GPCR involved in *Salmonella* engulfment by *Tetrahymena*. In order to deorphanize this receptor, we expressed its codon-optimized cDNA in a yeast heterologous expression system that exploits a histidine prototrophic phenotype upon GPCR activation by a cognate ligand (Kimber *et al.*, 2009; Wang *et al.*, 2006). We hypothesized that a *Tetrahymena* surface molecule activates the putative GPCR on neighboring *Tetrahymena* cells, thus we used *Tetrahymena* lysates as “ligands”.

We also assessed the activation of the putative GPCR in the presence of a suramin, a non-specific GPCR inhibitor (Freissmuth *et al.*, 1999).

**Fig. 4:**
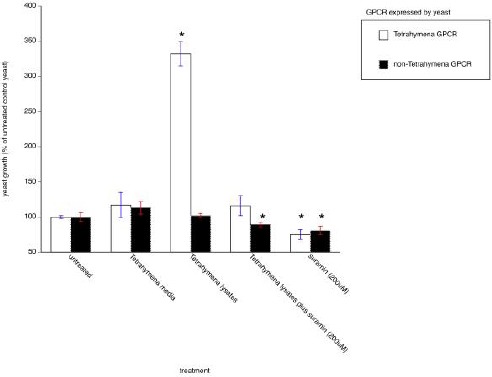
*Tetrahymena* lysate-mediated activation of histidine prototrophism in yeast expressing the *Tetrahymena* GPCR. Yeast transformants were exposed to the lysates and yeast growth was measured spectrophotometrically at OD_600_. To determine background growth, yeast transformants were grown in media lacking leucine and histidine (untreated control). As an additional control, yeast cells were transformed with a vector encoding an unrelated GPCR (from *C. parvum*) and then grown in the same conditions. Growth is quantitated as compared to growth observed in untreated controls. Data presented are the mean ± sem for three independent experiments each performed in triplicate. *p<0.05 versus untreated controls.

Treatment-control yeast were grown in leucine-deficient media in the absence of lysates or suramin. Transformation-control yeast were transformed with a vector encoding a non-*Tetrahymena* GPCR whose gene was cloned from *C. parvum*. As shown in Fig. 4, *Tetrahymena* lysates elicited significant increases in yeast growth in the transformants expressing the *Tetrahymena* GPCR. This growth was blocked by the addition of suramin. *Tetrahymena* lysates had no effect on yeast expressing the non-*Tetrahymena* GPCR cloned from *C. parvum*. However, suramin was able to reduce the basal activity associated with this non-*Tetrahymena* GPCR.

### Secondary structure analysis of the putative Tetrahymena GPCR

**Fig. 5:**
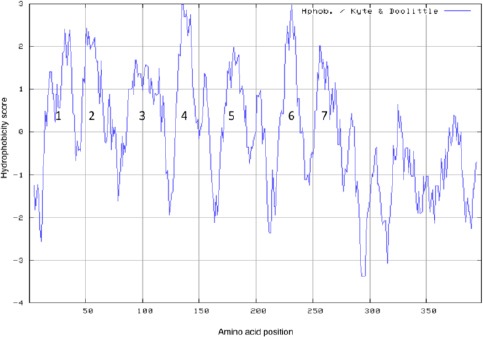
Kyte-Doolittle hydropathy plot of the putative *Tetrahymena* GPCR. Putative transmembrane domains were assessed using Kyte-Doolittle plots (Kyte and Doolittle, 1982) from a web-based platform (*http://web.expasy.org/cgi-bin/protscale/protscale.pl*). Transmembrane signatures were ascribed to peptide regions in which hydropathy scores rose from negative values to values greater than one. Putative transmembrane regions are indicated numerically from one to seven.

To assess the presence of motifs suggestive of a GPCR, a Kyte-Doolittle hydropathy plot (Kyte and Doolittle, 1982) was created for the putative *Tetrahymena* GPCR. Fig. 5 reveals that the putative *Tetrahymena* GPCR has the characteristic seven transmembrane domains.

## Discussion

The objective of this study was to identify protozoal determinants involved in the engulfment of *Salmonella*, a phenomenon that hyperactivates the virulence of the bacteria (Rasmussen *et al.*, 2005; Carlson *et al.*, 2007; Xiong *et al.*, 2010; Brewer *et al.*, 2011). Protozoa enhance the virulence of SGI1-bearing *Salmonella* by hyperactivating the expression of the pro-invasion gene designated as *hilA*, and this hyperexpression leads to enhanced virulence when the bacteria escape from the protozoa. The hyperactivation of *hilA* expression is dependent on the SGI1-specific gene designated as SO13 (Carlson *et al.*, 2007).

This phenomenon specifically occurs when SGI1-bearing *Salmonella* are engulfed by and then are liberated from free-living protozoa, such as *Acanthamoeba* and *Tetrahymena* (Carlson *et al.*, 2007), or from host-associated protozoa (Rasmussen *et al.*, 2005; Carlson *et al.*, 2007; Xiong *et al.*, 2010; Brewer *et al.*, 2011). The protozoa/SGI1-bearing *Salmonella* hypervirulence relationship appears to be relevant to the ingestion of water contaminated with free-living protozoa harboring SGI1-bearing *Salmonella* (Xiong *et al.*, 2010), and cattle that naturally harbor large numbers of protozoa in their rumen (Rasmussen *et al.*, 2005; Carlson *et al.*, 2007; Xiong *et al.*, 2010; Brewer *et al.*, 2011).

GPCRs were the focus of the present study since these receptors are apparently involved in *Tetrahymena* phagocytosis of particulates (Renaud *et al.*, 1995), and another group presented evidence for GPCR-encoding genes in the *Tetrahymena* database (Lampert *et al.*, 2011). Additionally, a GPCR was implicated in the engulfment of bacteria by the pathogenic protozoan *Entamoeba histolytica* (Picazarri *et al.*, 2005). In the present study we provide evidence that a putative GPCR governs *Salmonella* engulfment by *Tetrahymena*. It also appears that this engulfment process is dependent upon the density of protozoa, suggesting intercellular communication by *Tetrahymena*.

It appears that a specific seven transmembrane-spanning protein mediates the engulfment of *Salmonella*. This protein is putatively a GPCR given its ability to stimulate histidine prototrophism in a yeast heterologous expression in which G proteins govern *de novo* synthesis of histidine following ligand occupancy of a GPCR. Histidine prototrophism was observed in the presence of *Tetrahymena* lysates while suramin was able to block the histidine prototrophism in the presence of *Tetrahymena* lysates, further indicating that the protein is a GPCR. Suramin has some unknown effects, but we found that this molecule inhibited the *Tetrahymena* GPCR in two disparate systems.

Additionally, suramin reduced the basal activity of a non-*Tetrahymena* GPCR in the yeast system. Because of the unknown effects of suramin, however, we are unable to completely rule out GPCR-independent mechanisms contributing to its effects on *Tetrahymena* and the yeast.

Since *Tetrahymena* lysates activated the putative GPCR, an unknown *Tetrahymena* factor(s) appears to be an autocrine-like ligand for this receptor. This factor may be membrane-associated, whereby the receptor could be constitutively activated due to continual ligand occupancy when *Tetrahymena* are confluent. Alternatively, this may be a secreted factor. Although *Tetrahymena* has been established as an important model protozoan, there is little information on GPCRs in this organism except for a recent study (Lampert *et al.*, 2011). Interestingly, there has been debate regarding the existence of *Tetrahymena* GPCRs (Renaud *et al.*, 1991).

In conclusion, we determined that a putative *Tetrahymena* GPCR is responsible for the engulfment of *Salmonella* by the protozoan. We also partially characterized this unique receptor that appears to be activated by intercellular ligands whose threshold appears to be dependent upon the density of protozoa.
